# Symptomatic Squamous Papilloma of the Uvula: Report of a Case and Review of the Literature

**DOI:** 10.1155/2012/329289

**Published:** 2012-04-17

**Authors:** Lindsay A. Goodstein, Andleeb Khan, Joel Pinczewski, VyVy N. Young

**Affiliations:** ^1^University of Maryland School of Medicine, Baltimore, MD 21201, USA; ^2^Department of Otorhinolaryngology, University of Maryland, Baltimore, MD 21201, USA; ^3^Department of Pathology, University of Maryland, Baltimore, MD 21201, USA

## Abstract

*Background*. Oral squamous papillomas are benign pedunculated masses that grow most commonly on the palate. These benign lesions rarely cause symptoms. *Methods*. Here we present the case of a large, elongated squamous papilloma of the uvula causing dysphagia. We also review pertinent literature related to these lesions. *Results*. This patient underwent surgical excision of her atypically symptomatic oral lesion, with complete resolution of symptoms. *Conclusion*. Oral squamous papillomas are benign lesions which are usually asymptomatic. Dysphagia due to a squamous papilloma of the uvula has only been reported once in the literature previously. The development of symptoms such as dysphagia due to squamous papilloma of the uvula is uncommon; however this may be more likely in the presence of particularly large lesions.

## 1. Introduction

Squamous papillomas are exophytic masses of the oral cavity. When they occur on the palate, they are most often benign and asymptomatic [1–3]. Here we present the case of a squamous papilloma of the uvula that atypically produced symptoms.

## 2. Case Report

**Figure 1:**
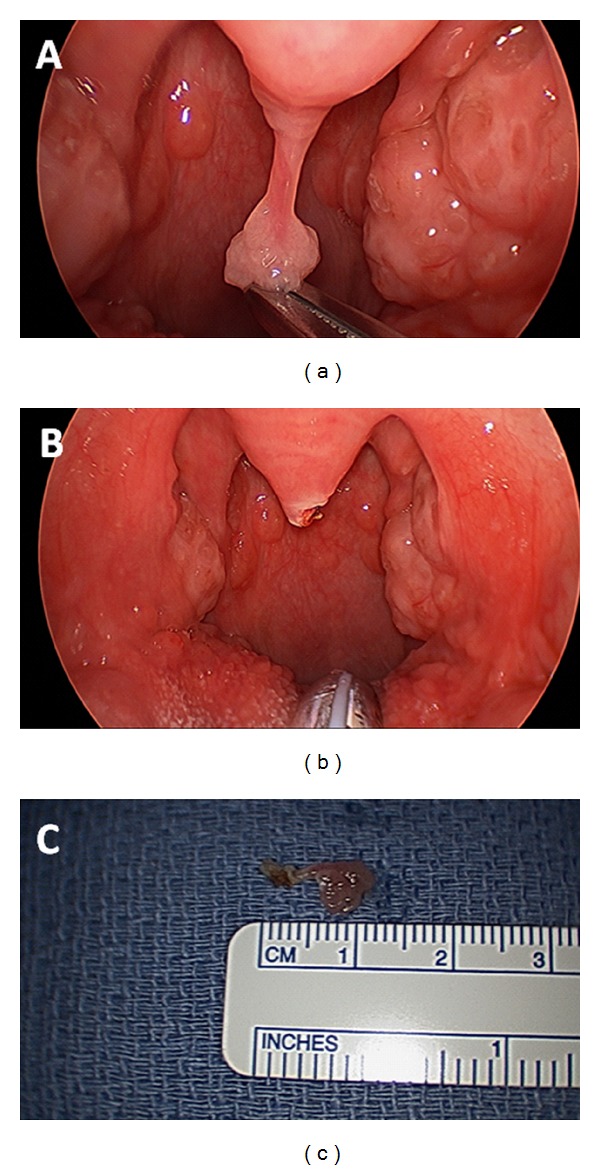
(a) Telescopic view of the pharynx reveals a pedunculated lesion emanating from the tip of the uvula. (b) Telescopic view of the pharynx after excision of the uvular lesion. (c) The squamous papilloma removed from the oral cavity.

A 22-year-old woman presented with 6–8 weeks of dysphagia. She also reported a choking sensation while eating food and brushing her teeth. She had a known history of uvular elongation, without change in size, for several years. She also complained of globus sensation, frequent throat clearing, dry cough, excess mucus, and heartburn. Examination revealed a pedicled lesion, approximately 1.5 cm in length, extending from the inferior tip of her uvula (Figure 1(a)).

**Figure 2:**
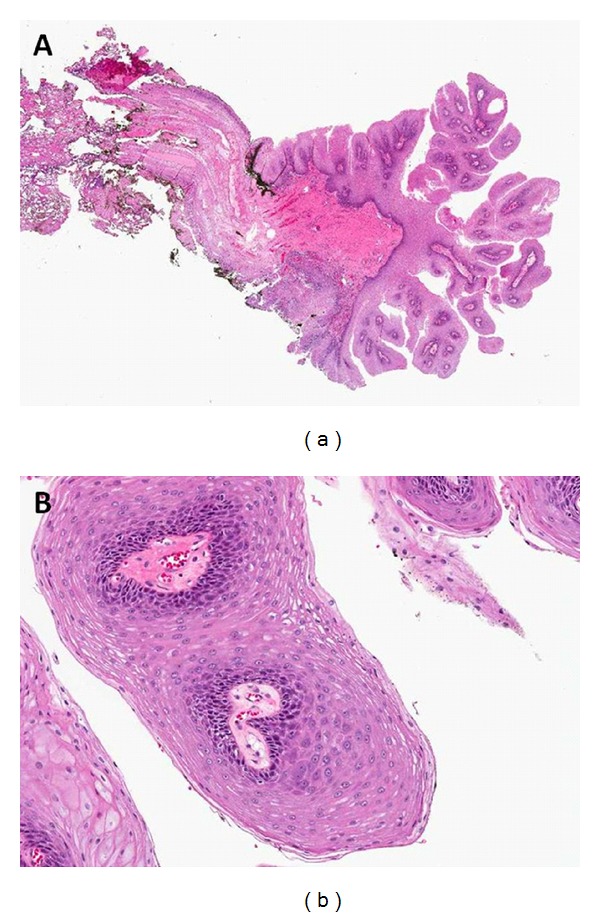
Low power (10×) and high power (50×) hematoxylin and eosin stained sections from the uvula lesion. (a) The low power view demonstrates a papillary lesion consisting of multiple squamous lined papillary fronds containing fibrovascular cores. (b) At higher power the squamous cells show bland histological features. These findings are characteristic of a squamous papilloma.

The lesion was completely excised from its connection to the uvula under general anesthesia with electrocautery (Figures 1(b) and 1(c)). Pathology revealed characteristic findings of a squamous papilloma, including multiple squamous lined papillary fronds containing fibrovascular cores (Figures 2(a) and 2(b)). The patient's postoperative course was unremarkable. Although she remains now on pharmacologic treatment for acid reflux, she reported complete resolution of all symptoms.

## 3. Discussion

Squamous papillomas are benign mucosal masses most commonly induced by HPV-6 and HPV-11. They typically present as single pedunculated masses with numerous finger-like projections at the surface [1]. Squamous papillomas are well described in the literature, with two cases of uvular papillomas reported as early as 1930 and 1931 [4, 5]. Reaching peak occurrence in adulthood, squamous papillomas develop most frequently on the palate and tongue [2]. In a case series of 464 oral squamous cell papillomas, 34.3% of cases were located on the palatal complex (hard, soft, and uvula), but only 4.2% of cases were located on the uvula [3]. One case of a squamous papilloma of the uvula interfering with swallowing has been reported in the literature [6]. The patient in that case, as well as the case presented here, had a papilloma greater than one cm in length, which occurs in less than 25% of cases of oral papillomas [3]. Therefore, it is probable that the greater the length of the uvular lesion, the more likely it is to become symptomatic. Unlike squamous papillomas of the larynx, squamous papillomas of the oral cavity are not associated with cancer and rarely recur. Surgical removal is the treatment of choice and can be performed with electrocautery, cold-steel excision, laser ablation, cryosurgery, or intralesional injections of interferon [7].
